# Ultrahigh-resolution nonlinear optical imaging of the armchair orientation in 2D transition metal dichalcogenides

**DOI:** 10.1038/lsa.2018.5

**Published:** 2018-05-04

**Authors:** Sotiris Psilodimitrakopoulos, Leonidas Mouchliadis, Ioannis Paradisanos, Andreas Lemonis, George Kioseoglou, Emmanuel Stratakis

**Affiliations:** 1 Institute of Electronic Structure and Laser, Foundation for Research and Technology-Hellas, Heraklion Crete 71110, Greece; 2 Department of Physics, University of Crete, Heraklion Crete 71003, Greece; 3 Department of Materials Science and Technology, University of Crete, Heraklion Crete 71003, Greece

**Keywords:** armchair orientation mapping, atomically thin transition metal dichalcogenides, crystal quality marker, graphene-related materials, polarization-resolved second-harmonic generation

## Abstract

We used nonlinear laser scanning optical microscopy to study atomically thin transition metal dichalcogenides (TMDs) and revealed, with unprecedented resolution, the orientational distribution of armchair directions and their degree of organization in the two-dimensional (2D) crystal lattice. In particular, we carried out polarization-resolved second-harmonic generation (PSHG) imaging for monolayer WS_2_ and obtained, with high-precision, the orientation of the main crystallographic axis (armchair orientation) for each individual 120 × 120 nm^2^ pixel of the 2D crystal area. Such nanoscale resolution was realized by fitting the experimental PSHG images, obtained with sub-micron precision, to a new generalized theoretical model that accounts for the nonlinear optical properties of TMDs. This enabled us to distinguish between different crystallographic domains, locate boundaries and reveal fine structure. As a consequence, we can calculate the mean orientational average of armchair angle distributions in specific regions of interest and define the corresponding standard deviation as a figure-of-merit for the 2D crystal quality.

## Introduction

The emerging family of graphene and related two-dimensional (2D) materials has provided researchers with fertile ground for exploring fundamental physical phenomena and developing innovative technological solutions^1, 2, 3, 4, 5, 6, 7^. Two-dimensional transition metal dichalcogenides (TMDs) MX_2_ (M: Mo or W, and X: S, Se, or Te) are direct bandgap semiconductors that are structurally similar to graphene^8^. These materials show electronic and optoelectronic properties, along with desirable mechanical strength and flexibility, that offer great promise for use in future electronic devices^9, 10, 11, 12, 13^.

While large-area crystal growth techniques, such as chemical vapor deposition (CVD), have been successfully used for the production of TMDs, they often fail in producing defect-free materials. As a consequence, the presence of grain boundaries, vacancies and differently oriented grains substantially affect the crystal quality, which is unavoidably reflected in the physical properties of the material^14, 15, 16^. At the same time, the presence of imperfections is undesirable for emerging applications for which large-scale production of high purity materials is essential. Currently, the study and evaluation of the crystal quality of TMDs is carried out using either complex and time-consuming methods or techniques that offer poor resolution. In particular, transmission electron microscopy (TEM) has been extensively used to monitor and map grain boundaries and crystallographic orientations with high sensitivity^17^. However, although TEM provides highly accurate crystal orientation information and atomic-scale resolution, the method is not suitable for large-area characterization. In addition, the material under study needs to be transferred from the underlying substrate to an electron-permeable TEM-supporting membrane, which is a time-consuming and invasive process that requires complex procedures for sample preparation. Consequently, there is currently no easily applicable, non-invasive and fast characterization method for determining, with high resolution and sensitivity, changes in crystallographic orientations as well as grain and other extended defect boundaries over a large 2D crystal area. Therefore, there is a need to develop quantitative metrics to quickly evaluate the quality of 2D crystals and provide direct feedback for process control during material growth.

Optical microscopy enables large-area imaging, but with moderate spatial resolution. In particular, polarized light microscopy provides quantitative information on the crystallographic symmetry of a material and can be used to locate defect or strained regions^18^. Nevertheless, it fails to provide sufficient intrinsic contrast for differentiating grain boundaries within a 2D atomic layer. Lately, nonlinear optical measurements, including second-harmonic generation (SHG) used in conjunction with laser scanning microscopy, have created new opportunities for improving the image resolution of 2D crystals^19, 20, 21, 22, 23, 24, 25, 26, 27^. Indeed, the SHG signal depends on the elements of the second-order susceptibility tensor *χ*^(2)^, which are non-vanishing only for non-centrosymmetric media such as the atomically thin TMDs^21, 28^. At the same time, the polarization of the SHG field depends crucially on the 2D crystal symmetry and orientation^22, 29, 30, 31^. Based on such SHG signal dependencies, the crystal quality of TMDs has been recently evaluated by several groups^21, 22, 25^. These studies consider a field of fixed polarization incident on a rotating sample, and upon measuring the SHG fields in the directions parallel and perpendicular to the fundamental, information on the domain boundaries and relative orientations of the crystals is obtained. While these methods enable the imaging of grain orientations with moderate contrast, the spatial resolution reported is insufficient to resolve information about crystallographic imperfections. An alternative method relies on using a stationary sample and rotating the polarization angle of the incident field, enabling imaging of the polarized SHG (PSHG) signal over an extended sample area.

In this work, we demonstrate a methodology to reveal crystallographic imperfections over large areas in 2D TMD crystals. It relies on rotating the polarization angle of the incident field with respect to a stationary sample and measuring the PSHG signal anisotropy for each individual pixel (pixel size of 120 nm) over a large crystal area. In particular, we fit, pixel-by-pixel, the PSHG data to a new generalized theoretical model that we developed, accounting for the characteristics of PSHG from 2D crystals. Based on this self-consistent process, we can accurately map the predominant crystal orientation, that is, the armchair direction, for each individual, 120 × 120 nm^2^, pixel in a specific region of interest (ROI) of the 2D crystal area. Consequently, we can calculate the standard deviation (*σ*_2D_) of the mean armchair orientation and provide a novel all-optical, quantitative quality factor for the structural characterization of 2D TMD crystals.

## Materials and methods

### Polarization-resolved SHG in stationary, raster-scanned 2D TMDs

**Figure 1:**
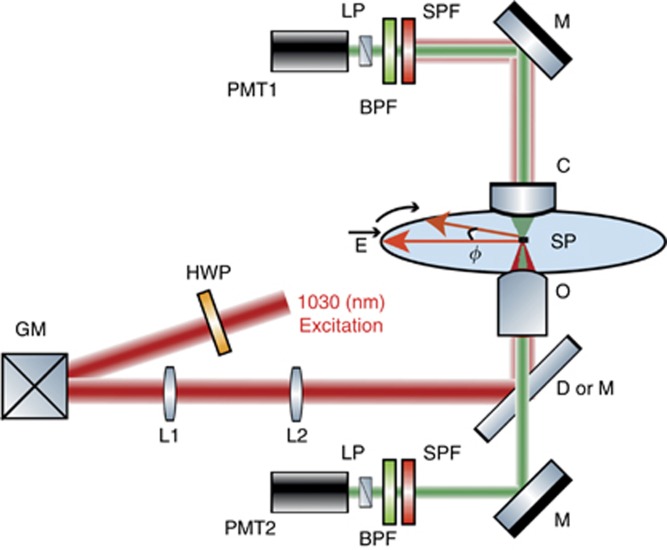
High-resolution, high-accuracy polarization-resolved SHG measurements. Abbreviations: BPF, bandpass filter of 514/3 nm for SHG in the transmission and 620/52 nm for 2PL in the epi-detection; C, condenser, 1.4NA; D or M, dichroic (for 2PL) or silver mirror (for PSHG), both at 45° GM, silver-coated galvanometric mirrors; HWP, zero-order half-waveplate; L1,2, achromatic lenses; LP, linear polarizer; M, silver mirror; O, objective 40 ×, 1.3NA; PMT1,2, photomultiplier tubes; SP, sample plane; SPF, short-pass filter. The excitation linear polarization starts horizontal in the SP and is rotated clockwise with an angle *φ*. Typically, *φ* is rotated 0°–360° with a step of 1°.

Combined PSHG and two-photon photoluminescence (2PL) imaging was carried out using a custom-built laser scanning microscope (Figure 1). Light from a diode-pumped Yb:KGW fs oscillator (1.2 W, 1030 nm, 70–90 fs, 76 MHz, Pharos-SP, Light Conversion) was inserted into an inverted microscope (Axio Observer Z1, Carl Zeiss, Jena, Germany) after passing through a pair of silver-coated galvanometric mirrors (6215H, Cambridge Technology, Bedford, MA, USA).

A motorized rotation stage (M-060.DG, Physik Instrumente, Karlsruhe, Germany), holding a zero-order half-wave retardation plate (QWPO-1030-10-2, CVI Laser), was used to control and rotate the orientation of the excitation linear polarization at the sample plane. Using a pair of suitable achromatic doublet lenses (forming a telescope), the beam emerging from the galvanometric mirrors was expanded approximately seven times in order to fill the back aperture of the objective lens.

Exiting the telescope, the beam was reflected on a silver-coated mirror, at 45° (PFR10-P01, ThorLabs, Newton, NJ, USA), placed at the motorized turret box of the microscope, just below the objective (Plan-Apochromat × 40/1.3NA, Carl Zeiss). Consequently, no dichroic mirror was used for our PSHG experiments. This choice, along with the silver coating on all of our mirrors (PF 10-03-P01, ThorLabs), including the galvanometric mirrors, rendered our setup insensitive to the laser beam polarization and its angle of incidence. The mean polarization extinction ratio of the different linear polarization orientations, calculated using crossed polarization measurements at the sample plane, was 28:1.

In the forward direction, immediately after the sample, the SHG signals were collected using a high numerical aperture (NA) condenser lens (achromatic-aplanatic, 1.4NA, Carl Zeiss). The SHG was separated from the laser using a short-pass filter (FF01-720/SP, Semrock, Rochester, NY, USA) and from the 2PL signals using a narrow bandpass filter (FF01-514/3, Semrock) before entering the forward detector, based on a photomultiplier tube (PMT) module (H9305-04, Hamamatsu, Hizuoka, Japan). A rotating film polarizer (LPVIS100-MP, ThorLabs) was inserted just in front of the PMT to measure the anisotropy due to the polarization of the SHG signals.

The 2PL signals were collected in the backward (epi-) detection geometry, with the same objective used for excitation and by using a short-pass dichroic mirror at 45° (DMSP805R, ThorLabs) placed at the motorized turret box of the microscope. The 2PL signals were separated from any laser light passing through the dichroic mirror using a short-pass filter (FF01-790/SP, Semrock) and from the SHG using a bandpass filter (MF620/52, ThorLabs). When the dichroic mirror was used, SHG imaging in the forward direction and 2PL imaging in the epi-detection were achieved simultaneously using a second PMT (H9305-04, Hamamatsu).

The galvanometric mirrors and the PMTs were connected to a connector block (BNC-2110, National Instruments, Austin, TX, USA), which was interfaced to a PC through a DAQ (PCI 6259, National Instruments). Coordination of PMT recordings with the galvanometric mirrors for the image formation, as well as the movements of all the microscope motors, was carried out using LabView (National Instruments) software.

The average laser intensity incident on the sample was ~3 MW cm^−2^. At this intensity level, no damage was observed for illumination periods as long as 3 h. With the above configuration, a maximum raster-scanned field of view (FOV) of 290 × 290 μm^2^ was obtained using the × 40, 1.3NA objective. Here, we chose to raster scan an FOV of 145 × 145 μm^2^, and each PSHG image was typically composed of 1200 × 1200 pixels, resulting in an acquisition time of ~3 s per frame and a pixel size of 120 × 120 nm^2^. The optical resolution in our experiment was 560 nm, as deduced by measuring the point spread function using SHG from 80-nm-diameter Au nanospheres. Compared to the theoretical resolution limit of 483 nm at 1030 nm, our PSHG setup performed close to the diffraction limit.

Note that since the thickness of the 2D monolayers (less than 1 nm) is orders of magnitude smaller than the axial dimension (hundreds of nm) of the focused spot in our setup, common experimental errors in PSHG microscopy, such as birefringence and polarization scrambling due to scattering, are minimized.

### Extraction of the armchair symmetry axis *θ* of WS_2_

**Figure 2:**
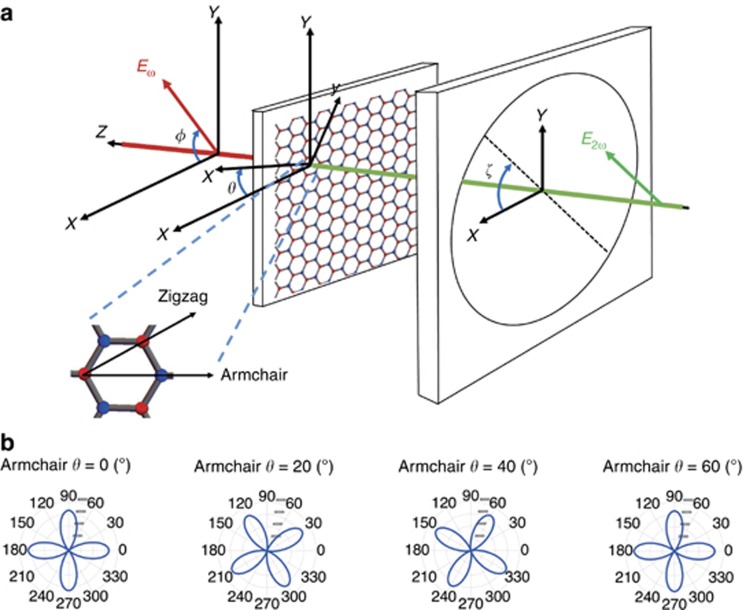
(**a**) Coordinate system describing the experimental measurements and introducing relevant angles with respect to the laboratory frame; *φ*, angle between excitation linear polarization *E*_ω_ and *X*-laboratory axis; *θ*, angle between TMD armchair crystal orientation -*x* and the *X*-reference axis; *ζ*, angle between linear polarizer axis and *X*-axis. The crystal structure and symmetry axis of WS_2_ are shown (top view) magnified. (**b**) Simulation of the PSHG intensity modulation as a function of the linear excitation angle *φ*, described by Equation (1), for four different armchair orientations, *θ*=0°, 20°, 40° and 60°, when *ζ*=0. Note that for a fixed position of the analyzer, the fourfold symmetric polar diagram of the SHG intensity rotates as a function of the armchair crystal orientation.

In its bulk form, the WS_2_ crystal comprises two honeycomb sublattices—one comprising W atoms and the other S atoms—shifted relative to each other; therefore, 2H-WS_2_ is inversion symmetric (for an even number of layers), belonging to the D_6h_ symmetry group, characteristic of the Bernal-stacked trigonal prismatic structure. However, this symmetry is broken for an odd number of layers due to stacking termination along the *c*-axis, resulting in non-centrosymmetric structures. In the case of one layer, an individual layer of W atoms with threefold symmetry is hexagonally packed between two trigonal atomic layers of S atoms, resulting in a hexagonal honeycomb lattice (top view in Figure 2a), where the W and S atoms are located at alternate corners of the hexagon. Therefore, the WS_2_ monolayer belongs to the D_3h_ point symmetry group, with broken inversion symmetry along the armchair direction. The lack of inversion symmetry in the monolayer results in SHG when an intense field is incident on the crystal.

In Figure 2a, the experimental configuration is presented, exhibiting two relevant coordinate systems (laboratory *X*-*Y*-*Z* and crystalline *x*-*y*-*z*) along with the various angles involved. The laser beam is normally incident on the sample plane at an initial excitation linear polarization angle of *φ* with respect to the laboratory frame (*X*, *Y*, *Z*). The armchair direction points along the *x*-axis of the crystalline coordinate system (*x*, *y*, *z*). The latter is rotated by an unknown (but constant) angle *θ* with respect to the laboratory *X*-axis. The SHG signal generated by the WS_2_ crystal following interaction with the incident laser is passed through the analyzer (linear polarizer), which is at an angle *ζ* with respect to the laboratory frame, before reaching the detector.

As mentioned above, a hexagonal lattice is created by alternating W and S atoms at the corners of the hexagon, resulting in a non-centrosymmetric structure for an odd number of layers. The magnified crystal lattice in Figure 2a shows the atom configuration in the hexagon along with the armchair direction (which defines the mirror symmetry axis) and the zigzag direction (which is 30° from the armchair and connects atoms of the same element). Note that the WS_2_ flakes show two different edge terminations: tungsten zigzag and sulfur zigzag, which are the two most energetically stable edge orientations^32^.

The theoretical dependence of the SHG intensity on the relative orientation of the input laser polarization and lattice orientation has been previously reported for different types of bulk crystals^33, 34^. Here, we extend this idea to 2D materials of the TMD family while at the same time, we generalize the equations to account for both *θ*- and *φ* -dependencies. Under the D_3h_ symmetry characterizing the WS_2_ crystal, only the following four elements of the second-order susceptibility tensor are nonzero: 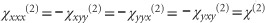. If we apply the theory of nonlinear SHG^35^ (see Supplementary Materials for a detailed derivation), we obtain the following formula describing the total SHG intensity detected by the PMT:


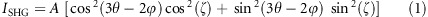


where A is a multiplication factor depending on the amplitude of the excitation field and the SHG susceptibility tensor *χ*^(2)^ of the material. For the analyzer orientations *ζ*=0 and *ζ*=*π*/2, we obtain the two normal components of the SHG field, that is, *I*_*X*_~cos^2^(3*θ*−2*φ*), and *I*_*Y*_~sin^2^(3*θ*−2*φ*), respectively.

The graphical representation of Equation (1) at a fixed analyzer position of *ζ*=0 and the corresponding visualization in a polar diagram (Figure 2b) demonstrates a fourfold symmetry for the SHG intensity that rotates for different armchair orientations *θ* (for different armchair angles and choice of *ζ*=*π*/2, see also the polar diagrams in Supplementary Fig. S2 of Supplementary Materials). Thus, each armchair orientation corresponds to a characteristic fourfold symmetric polar diagram. Consequently, by fitting pixel-by-pixel the PSHG experimental data to Equation (1), we can acquire the armchair angle *θ* for every pixel of the image. We note that the armchair directions differing by 60° produce the same polar diagrams (that is, the armchair direction can be determined modulo 60°), reflecting the threefold rotational symmetry of the WS_2_ crystal (that is, the fact that there are three equivalent armchair axes). Furthermore, the excitation polarization for *φ* between 0° and 90° provides the same PSHG intensity modulation as for *φ* between 90° and 180°. This is also the case for *φ* between 180° and 270° or 270° and 360° (fourfold symmetry). Therefore, *φ* sampling in the range 0°–90° is adequate to retrieve all possible armchair orientations, that is, *θ*∈[0°–60°].

### Samples

The WS_2_ samples were grown by the low-pressure chemical vapor deposition method (LP-CVD) on a *c*-cut (0001) sapphire substrate (2D semiconductors). The samples were characterized using micro-Raman spectroscopy with a 473 nm excitation wavelength. A_1g_ mode intensity mapping was utilized to determine thickness variations across the sample (for more details, see Section 3 in Supplementary Materials and Supplementary Figs. S6 and S7).

## Results and discussion

### The PSHG contrast mechanism

Unlike previous experiments in which samples are rotated^21, 22, 25^, in this work, we keep the WS_2_ crystals fixed and rotate the polarization of the fundamental field with respect to the laboratory *X*-axis; in this way, we can record PSHG measurements from each point of a large raster-scanned surface area. This enables pixel-by-pixel evaluation of the crystal mirror symmetry axis (that is, the armchair direction)^21, 22^. For this purpose, the (unknown but constant) angle between the armchair direction and the laboratory *X*-axis is denoted here as *θ*, while *φ* is defined as the angle between the latter and the fundamental field (see Figure 2a and Supplementary Fig. S1).

**Figure 3:**
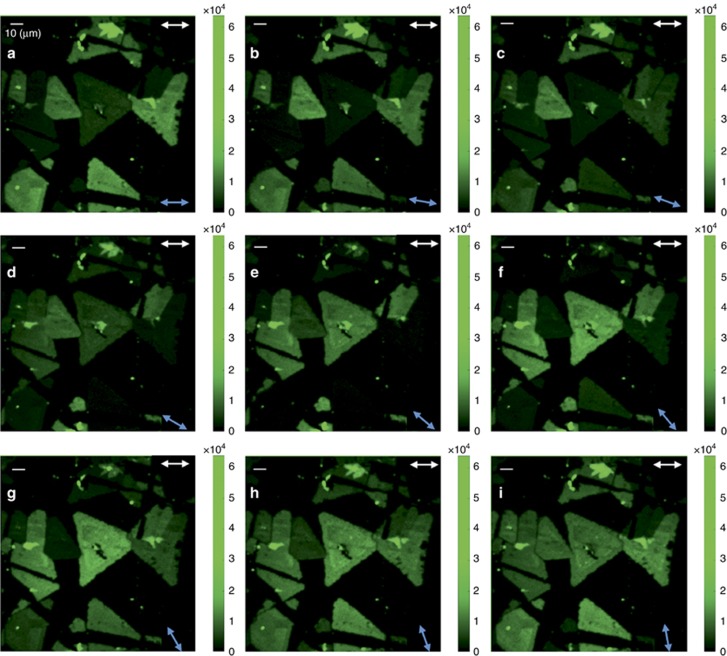
Forward detected polarization-resolved SHG intensity imaging of WS_2_ islands on a sapphire substrate (brighter color indicates higher PSHG intensity). Double arrows show the orientation *ζ* of the analyzer (white arrows, upper right) and the orientation *φ* of the linear excitation (blue arrows, lower right). (**a**)-(**i**) Rotation of excitation linear polarization orientation *φ* ∈[0°–80°], with a step of 10°, shows the switching on and off of the SHG signal from different triangular flakes depending on their relative armchair orientation *θ*. The scale bar is 10 μm (upper left).

**Figure 4:**
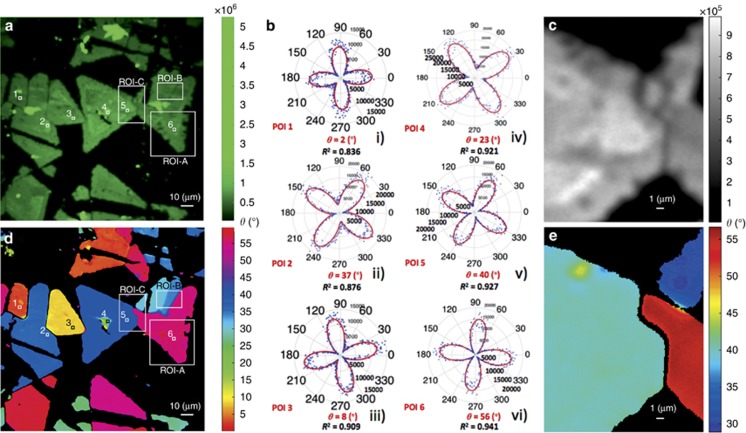
PSHG contrast mechanism based on mapping of armchair crystal orientations. (**a**) Integration of the PSHG data presented in Figure 3 for *φ* ∈[0°–90°] and a step of 1°. Regions of elevated SHG signal indicate thicker material. Six POIs are additionally marked. (**b**) The experimentally retrieved polar diagrams of SHG intensity as a function of the polarization angle (*φ* ∈[0°–360°], step of 1°) for the six POIs. The rotation of the four-leaved rose corresponding to different armchair orientations *θ* is evident. (**c**) Magnified view of ROI-C SHG intensity. (**d**) Mapping of armchair orientations *θ* over a large sample area. The POIs shown correspond to the same positions as in **a**. Two different ROIs, that is, ROI-A and ROI-B, are also indicated. (**e**) Mapping of armchair orientations *θ* over ROI-C.

In particular, by using a 1° step for the orientation of the excitation linear polarization, *φ*, monitoring of the PSHG signal with high accuracy was realized. Figure 3 shows typical PSHG snapshots of raster-scanned WS_2_ crystals while the angle *φ* was varied between 0° and 80° with a step of 10° for each consecutive image. It can clearly be observed that the rotation of *φ* gives rise to significant variations in the SHG signal intensity from different triangular flakes (in some flakes, it switches on and off), depending on their relative armchair crystal orientation *θ* (see also movie 1 in Supplementary Materials for *φ* between 0° and 360°, step of 1°). Figure 4a shows the integration of the PSHG images for *φ*∈[0°–90°], with a 1° step. In contrast to Figure 3, where the SHG signal changes with respect to *φ*, the integration of the PSHG images is no longer polarization dependent. Note that in the case of the experiment being carried out without using an analyzer, the measured SHG intensity would be identical to that shown in Figure 4a. Therefore, the use of the analyzer is advantageous in our method, as it enables the extraction of additional information regarding the crystal symmetry. When the dependence of the SHG intensity on the armchair angle *θ* is lost, any differences in intensity seen in Figure 4a can be solely attributed to thickness variations. Larger (odd) numbers of stacked monolayers generate an SHG signal of higher intensity in the 2H stacking sequence^21^; however, there is a possibility of an arbitrary 3R stacking order due to growth that will give rise to higher SHG intensity regardless of the number of stacked layers. Such monolayer versus multilayered areas are distinguishable in Figure 4a; indeed, some triangles show a uniform SHG signal and, thus, single layer thickness, whereas others reveal thickness variations with higher plateaus near their central areas. This is further confirmed via Raman spectroscopy mapping of the same areas (see also Supplementary Fig. S7 and the relevant discussion in Supplementary Materials).

**Figure 5:**
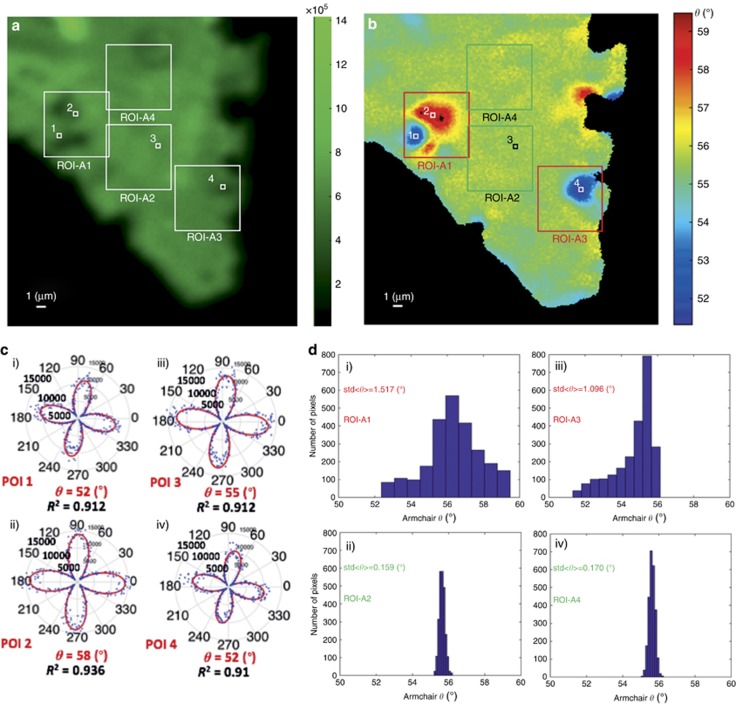
PSHG as a crystal quality marker: (**a**) Integrated PSHG for *φ* ∈[0°–90°], step of 1°, for ROI-A shown in Figure 4a. Four ROIs of 50 × 50 pixels, namely, ROI-A1, ROI-A2, ROI-A3 and ROI-A4, and four POIs are indicated. (**b**) Armchair mapping reveals grains of different crystal orientations, not seen in the SHG intensity image. (**c**) Experimental PSHG modulation (*φ* ∈[0°–360°], step of 1°) for POIs 1–4, fitted with Equation (1). (**d**) Image histograms showing the distribution of armchair orientations inside ROIs-A1-4. The crystal quality is reflected in the standard deviation (*σ*_2D_) of the mean armchair direction, <*θ*>. The grains of different crystal orientations seen in ROIs 1 and 3 gave *σ*_2D_ values equal to *σ*_2D_=1.517° and *σ*_2D_=1.096°, respectively, while in ROIs 2 and 4, the uniform distribution of armchair angles resulted in smaller *σ*_2D_ values, that is, *σ*_2D_=0.159° and *σ*_2D_=0.17°, respectively. Small *σ*_2D_ values are indicative of good crystal quality.

**Figure 6:**
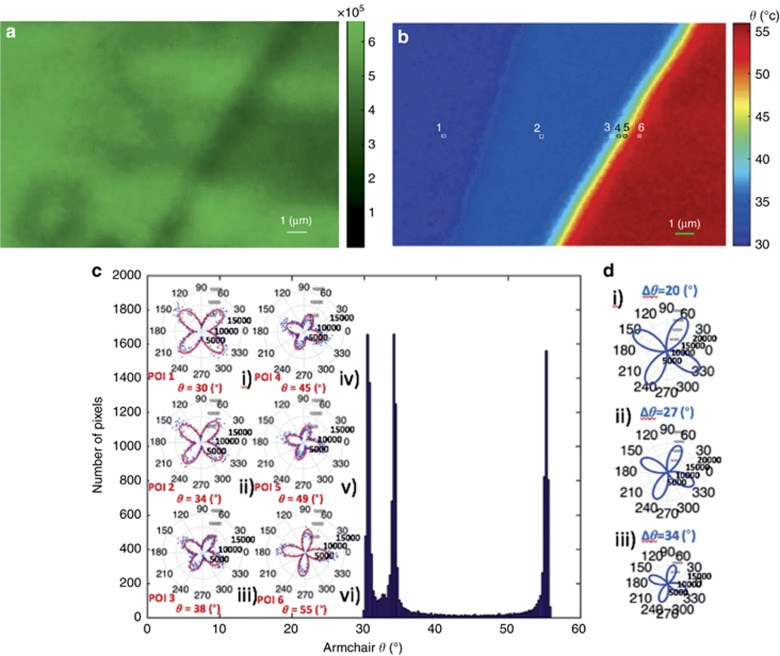
Boundaries between areas with different armchair directions. (**a**) Sum of SHG for *φ* ∈[0°–90°], step of 1°, for ROI-B seen in Figure 4a. (**b**) Armchair mapping reveals boundaries of different crystal orientations, not seen in the intensity-only SHG image. (**c**) Image histogram showing the distribution of armchair orientations; (i)-(vi) Experimental PSHG modulation [0°–360°] using a step of 1° for POIs 1–6, respectively, fitted with Equation (1). (**d**) Theoretical simulation of the PSHG intensity modulation at the boundary between crystals of different armchair orientations (see Equation S15 in Supplementary Materials). The theoretical prediction fits well with the experimental data shown in Figure 6c, that is, the four-leaved pattern rotates counterclockwise and its amplitude decreases.

To further explore the imaging capabilities of the proposed technique, we focus on specific pixels of interest (POIs) belonging to different triangles. As mentioned above, by plotting the SHG intensity dependence on the polarization angle *φ* in a polar diagram and fitting with Equation (1), we can determine the armchair angle *θ* for each individual pixel. For instance, the polar diagrams shown in Figure 4b show the intensities for the six different POIs shown in Figure 4a, together with the angles defining the corresponding armchair directions and the quality of the fitting factor, namely, *R*^2^. Following the same procedure for every single pixel in the image, we obtain Figure 4c, which is a color map of the armchair directions over a large sample area. Consistent with the theoretical prediction, we observe that the value of the armchair angle is between 0° and 60°. Although similar maps have been reported before^25^, our technique identifies the changes in armchair orientation and reveals extended crystallographic imperfections with much higher resolution (pixel dimensions: 120 × 120 nm^2^). Note that the 120 × 120 nm^2^ pixel size does not correspond to the optical resolution of our PSHG microscope, which was measured to be 560 nm, but rather to the resolution in the armchair imaging. Indeed, owing to the high contrast of our method, we are able to determine variations in the armchair orientation within areas of radius equal to 560 nm. This high resolution is realized due to the pixel-wise fitting procedure, enabling the discrimination of features beyond the optical resolution, in the ‘polarization space’^36^. Indeed, although the color mapping shows that each flake is characterized by its own main crystallographic axis (armchair), when reducing the scale of *θ*, specific regions within each flake appear with discontinuous coloration (compare ROI-A in Figure 4c with Figure 5a and 5b and with Supplementary Figs. S5g and S5h, S6d and S6e). Further inspection of the armchair maps shows that at the boundary between neighboring flakes, the armchair direction changes either continuously (see, for example, ROI-B in Figure 4c and the discussion related to Figure 6a and 6b below) or abruptly (see flakes containing POIs 1 and 2 in Figure 4c and Figure 4d and 4e). The latter is a manifestation of destructive SHG interference and occurs when suitable conditions are satisfied between the neighboring armchair directions (see the relevant discussion below and Section 2 of Supplementary Materials). It should be emphasized here that all the structural details revealed by our method and described above cannot be resolved, with such a high sensitivity, via conventional spectroscopy, including Raman and 2PL imaging (see supplementary Figs. S4-S7). Notably, the smallest angle change that is experimentally detectable with our technique for an ROI of 50 × 50 pixels is ~0.16° (Figure 5dii), in excellent agreement with the numerical prediction of ~0.19° for an individual pixel (see the calculation in Supplementary Materials).

A closer examination of the high-resolution armchair orientation maps reveals that crystal areas that initially appeared to feature a uniform armchair direction include characteristic domains deviating from such uniformity. A typical example is the region ROI-A indicated in Figure 4a, magnified by ten times in Figure 5. Inside this ROI, we chose four additional ROIs: ROI-A1, ROI-A2, ROI-A3 and ROI-A4, each of 50 × 50 pixels in size (Figure 5a and 5b), which include typical examples of variations in armchair directions. Such variations are reflected in the different polar diagrams shown in Figure 5c, which were obtained from the four pixels indicated in Figure 5a and 5b, (POI-1 and POI-2 are from ROI-A1, POI-3 from ROI-A2 and POI-4 from ROI-A3). The respective image histograms showing the distribution of armchair orientations inside ROI-A1-4 are shown in Figure 5d. It is clear that ROIs showing large variations in armchair angles, that is, extensive crystallographic imperfections, are characterized by a larger standard deviation of armchair orientations, *σ*_2D_ (for example, ROI-A1 and -A3). This is in contrast to ROIs featuring small *σ*_2D_ values (for example, ROI-A2 and -A4). Based on these findings, we can define *σ*_2D_ as a quality factor for as-grown 2D crystals. Indeed, crystals of high quality are expected to have uniform armchair maps and narrow orientation distributions, giving rise to lower *σ*_2D_ values. It can be concluded that the high-contrast method we developed enables all-optical detection of 2D crystal imperfections that are otherwise invisible to conventional SHG intensity microscopy.

To further elucidate the experimental findings, we have focused on the boundary between adjacent regions exhibiting different predominant armchair orientations. Figure 6a shows the integrated SHG signal for *φ* ∈[0°–90°] with a step of 1° for the ROI-B seen in Figure 4a. The corresponding armchair orientation mapping shown in Figure 6b exhibits a gradual change in the armchair angle as one moves from the left (dark blue) region to the right (dark red) region. This contrast is not available in intensity-only SHG, Figure 6a.

Figure 6c shows the respective polar diagrams of the PSHG intensity, obtained from six differently colored pixels (POIs 1–6) inside the map shown in Figure 6b. As seen, the corresponding armchair angle values for each pixel are ~30°, 34°, 39°, 45°, 49° and 55°, respectively, while the histogram of the entire map comprises three dominant armchair orientations (exhibiting three narrow peaks at 30°, 34° and 55°). The intermediate values seen for the POIs 3-5 of Figure 6c are due to SHG interference, described by Equation S15 in Supplementary Materials. Nevertheless, the SHG signal does not reduce to zero in this interference region. Such a smooth variation in armchair orientation (that is, no presence of a dark interface) suggests that either the conditions for destructive interference are not satisfied for the selected neighboring flakes (these conditions are presented in Supplementary Materials) or that there exist edge-induced SHG signals at the grain boundaries^19^. A third possibility is that the SHG signal is due to an imbalance in the population of different valleys in this multi-valley electronic system^37^. In any case, when only the intrinsic nonlinear response of the crystal is considered, a grain boundary can give rise to either constructive or destructive interference, depending on the relative orientation between the neighboring regions of different armchair orientations^20, 27^. It should be emphasized here that, to date, discrimination between boundaries of different armchair orientations using SHG imaging was carried out in the absence of the signal due to destructive interference^20^, which is independent of the orientation of the excitation polarization (*φ*). Consequently, the identification of small variations in the angle *θ* was not possible^19^. Here, the pixel-wise polarization analysis of the SHG signal enables, for the first time, such discrimination of boundaries between areas exhibiting small differences in armchair orientation, even in the absence of the destructive interference effect (compare, for example, Figure 6a with Figure 6b). To provide further insight into the above findings, the theoretically calculated PSHG intensity (via Eq. S15) at the boundary between regions with different dominant armchair angles is polar plotted in Figure 6d as a function of the polarization angle (see also Supplementary Fig. S3 in Supplementary Materials). We have used three sets of *θ*_1_ and *θ*_2_ values by arbitrarily selecting the armchair direction of neighboring grains. It can be seen that upon increasing the angle difference Δ*θ*=*θ*_1_−*θ*_2_, the SHG intensity drops and the four-leaved pattern rotates counterclockwise. This is in qualitative agreement with the experimental findings of Figure 6c.

## Conclusions

We have demonstrated an all-optical, fast, and non-invasive method to resolve, with unprecedented resolution, the crystalline integrity of atomically thin WS_2_ crystals via experimentally probing and theoretically interpreting their nonlinear optical properties. In particular, we showed that PSHG raster-scanning imaging microscopy enables high-resolution pixel-by-pixel (pixel size of 120 × 120 nm^2^) mapping of the armchair crystal axis orientation, which can be used to provide first-order information on crystallographic imperfections stemming from inhomogeneities in the distribution of armchair orientations. Moreover, the measured pixel-by-pixel PSHG data were fit to a new theoretical model based on the nonlinear optical response of such crystals, which was utilized as a second-order filter that effectively enhances the optical contrast and provides quantification of the crystal quality in terms of the standard deviation of the calculated armchair orientational distributions. Consequently, we demonstrate an ultrahigh-resolution nonlinear optical method that can be used to identify small variations in the crystal armchair direction. This information with such a high resolution is unattainable by conventional SHG imaging as well as by Raman and photoluminescence mapping.

We envisage that this work will have significant impact on the study of atomically thin TMD crystal growth and will prove useful for the development of large-area defect-free atomically thin 2D materials with excellent optoelectronic properties.

## Author contributions

SP, ES and GK planned the project; SP, LM and IP designed the experiment; SP and IP conducted the optical experiments; SP, AL and LM conducted the data analysis; AL provided technical support; LM elaborated the theoretical model and the group theory analysis; ES and GK guided the research. All authors contributed to the discussion and preparation of the manuscript.
