# Supplementary material for: Ultrahigh-resolution nonlinear optical imaging of the armchair orientation in 2D transition metal dichalcogenides
Source: Light Sci Appl. 2018 May 4;7:18005–. doi: 10.1038/lsa.2018.5 (PMC6060071; doi:10.1038/lsa.2018.5)
Supplement: Supplementary Material [file lsa20185x1.docx]

**Supplementary Information for**

**Ultrahigh-resolution non-linear optical imaging of armchair orientation in 2D transition metal dichalcogenides**

Sotiris Psilodimitrakopoulos^1^, Leonidas Mouchliadis^1^, Ioannis Paradisanos^1,2^, Andreas Lemonis^1^, George Kioseoglou^1,3^ and Emmanuel Stratakis^1,3^

^1^Institute of Electronic Structure and Laser, Foundation for Research and Technology-Hellas, Heraklion Crete 71110,Greece

^2^Department of Physics, University of Crete, Heraklion Crete 71003, Greece

^3^Department of Materials Science and Technology, University of Crete, Heraklion Crete 71003, Greece

Correspondence: E. Stratakis, E-mail: [stratak@iesl.forth.gr](mailto:stratak@iesl.forth.gr);

1. **Theoretical model of PSHG**

In our experiment the angle between the crystal x-axis (the armchair direction) and the lab X-axis is constant and the polarization angle of the incident wave with respect to the X-axis is varied. The corresponding angles are shown in the following figure:


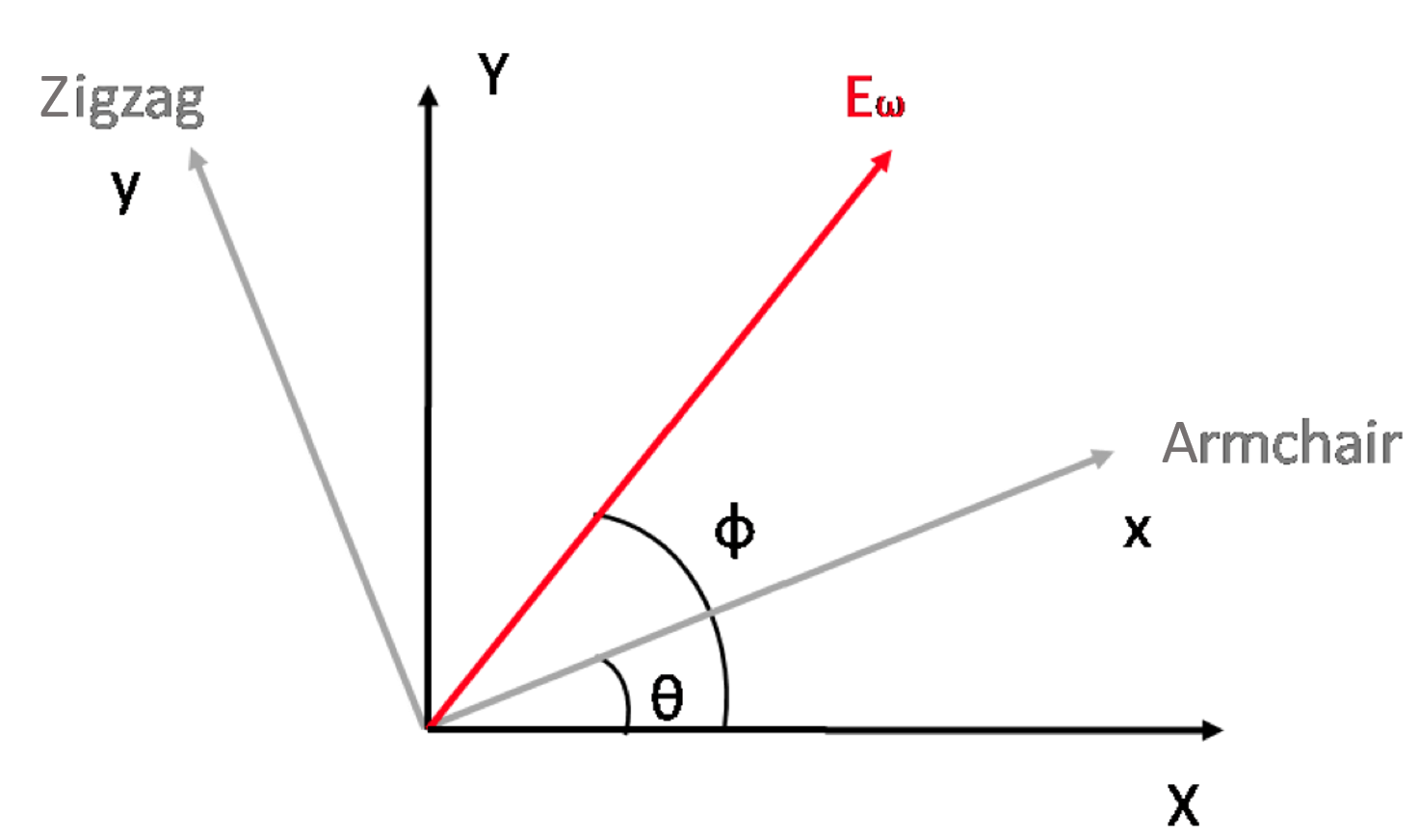


**Figure S1:** Coordinates system describing the experimental measurements and introducing relevant angles

Here, X and Y refer to the lab coordinates, x and y are the crystal coordinates, *θ* is the (constant) angle between the X-axis and the armchair direction and needs to be determined. $\varphi$ is the angle between the incident wave polarization and the lab X-axis which is what we control in the experiment.

The induced nonlinear polarization that leads to second harmonic generation in crystals can be described by the following equation:

$P_{i}\left( 2\omega\right)= \varepsilon_{0}\sum_{j, k} \chi_{ijk}^{\left( 2 \right)}E_{j}\left( \omega\right)E_{k}\left( \omega\right).$(S1)

Here, $\chi_{ijk}^{\left( 2 \right)}$ is a third rank susceptibility tensor which describes the symmetry properties of the crystal. In the case of D_3h_ point symmetry the above Eq. S1 written in matrix notation assumes the form

$\left( \begin{matrix} P_{x}^{2\omega} \\ P_{y}^{2\omega} \\ P_{z}^{2\omega} \end{matrix} \right)$ = $\varepsilon_{0}\chi_{xxx}^{(2)}\left( \begin{matrix} 1 & -1 & 0 & 0 & 0 & 0 \\ 0 & 0 & 0 & 0 & -1 & -1 \\ 0 & 0 & 0 & 0 & 0 & 0 \end{matrix} \right)\left( \begin{matrix} E_{x}^{\omega}E_{x}^{\omega} \\ E_{y}^{\omega}E_{y}^{\omega} \\ E_{z}^{\omega}E_{z}^{\omega} \\ {2E}_{y}^{\omega}E_{z}^{\omega} \\ {2E}_{x}^{\omega}E_{z}^{\omega} \\ 2E_{x}^{\omega}E_{y}^{\omega} \end{matrix} \right)$(S2)

The fundamental field is normal incident on the sample plane and has the form

$\boldsymbol{E}_{\boldsymbol{\omega}}=\cos\varphi\boldsymbol{i}+\sin\varphi\mathbf{j}$(S3)

Applying equation S2, we finally obtain the following formula for the SHG polarization field:

$\boldsymbol{P}_{\boldsymbol{2}\boldsymbol{\omega}}\sim cos[ 2(\varphi-\theta)]\boldsymbol{i}^{\boldsymbol{'}}-\sin[2(\varphi-\theta)]\mathbf{j}^{\mathbf{'}}$(S4)

where$\boldsymbol{i'}$and$\boldsymbol{j'}$are the unit vectors along x and y respectively and are connected with the unit vectors of the laboratory coordinate system via:

$\boldsymbol{i}^{\boldsymbol{'}}\boldsymbol{=}\cos\theta\boldsymbol{i+}\sin\theta\boldsymbol{j}\boldsymbol{j}^{\boldsymbol{'}}\boldsymbol{=}-sin \theta\boldsymbol{i+}\cos\theta\boldsymbol{j .}$(S5)

Substituting into the equation for the SHG polarization field we finally obtain:

$\boldsymbol{P}_{\boldsymbol{2}\boldsymbol{\omega}}\sim\cos[3\theta-2\varphi] \boldsymbol{i} +sin[ 3\theta-2\varphi]\boldsymbol{j}$(S6)

where we have used the following trigonometric identities:

$\sin\left( a-b \right)=\sin a\cos b-\cos a\sin b and cos \left( a-b \right)= \sin a\sin b-\cos a\cos b.$(S7)

Finally, the intensity of the PSHG for parallel and perpendicular positions of the analyzer are respectively:

$I_{x} \sim\cos^{2}[3\theta-2\varphi]$ and $I_{y} \sim\sin^{2}[3\theta-2\varphi]$(S8)


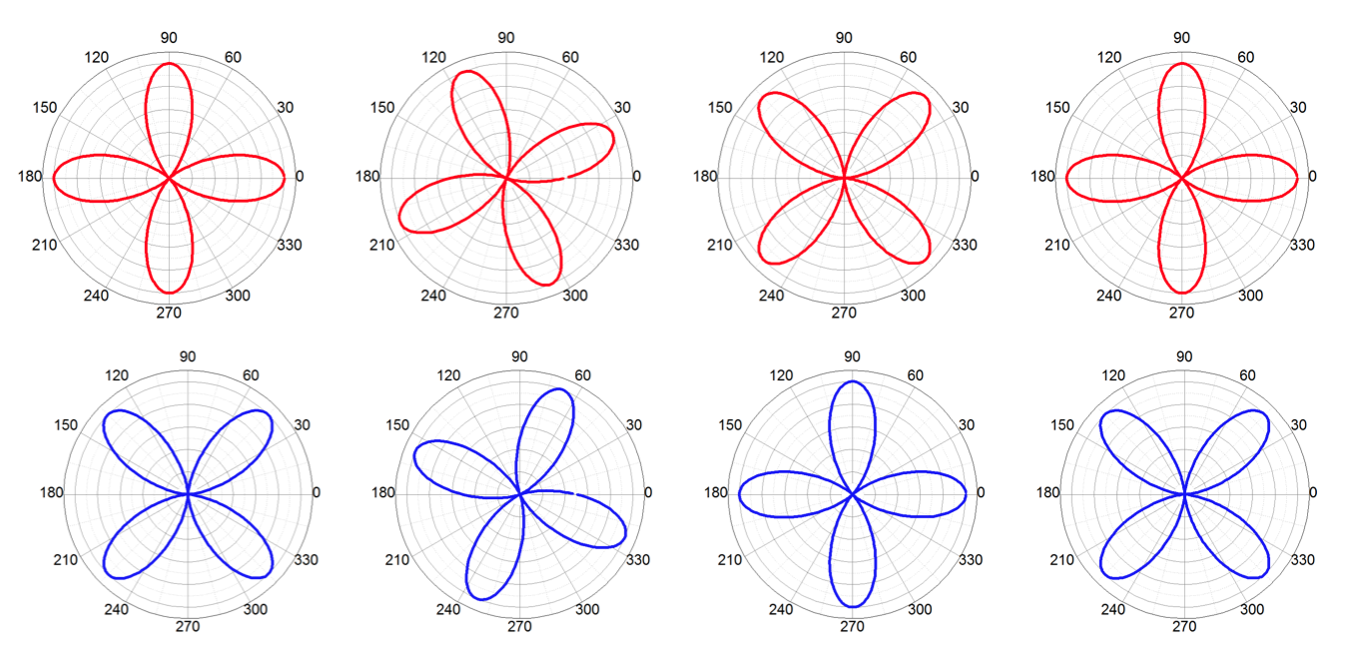


**Figure S2:** Polar diagrams of the SHG intensity as a function of the polarization angle for *θ*=0^o^, 15^o^, 30^o^ and 60^o^. The red plots correspond to the parallel component of the SHG field whereas the blue curves are the perpendicular ones.

The formula used for the fitting of the SHG intensity data for parallel position of the analyzer is:

$I_{SHG} = {A cos}^{2}[3\theta-2\varphi]$ +Δ (S9)

where *A* is a multiplication factor and *Δ* accounts for experimental errors introduced by the optical components of the setup, like constant background, ellipticity in the excitation polarization and ellipticity in the detected SHG polarization [1,2].

From the experimental data and the polar diagram, we can determine the angle between the crystal x-axis and the lab X-axis, i.e. the crystal orientation (armchair) *θ*. Alternatively, it is straightforward to derive the following expression which can be used to find *θ* if we substitute the measured I_x_ and I_y_ for a given polarization angle φ:

$\theta= \frac{1}{3}\left[ 2\varphi+\tan^{-1} \sqrt{\frac{I_{y}}{I_{x}}} \right]$(S10)

**2. Interference**

Neighboring triangular WS_2_ islands are characterized by differences in the main crystallographic axis which are encoded using the SHG imaging in the different colors that represent their armchair directions (see Fig. 6 in main text). Exactly at the boundaries of these regions the SHG might cancel when suitable conditions apply. In this subsection we investigate these conditions by applying a simple model of interfering waves. According to our generalization two different regions where the predominant armchair directions are *θ_1_* and *θ_2_* respectively, produce the following SH signals:

$\mathbf{P}_{2\omega,1}\sim\cos(3\theta_{1}-2\varphi)\boldsymbol{i} +\sin(3\theta_{1}-2\varphi) \mathbf{j}$(S11)

$\mathbf{P}_{2\omega,2}\sim\cos(3\theta_{2}-2\varphi)\boldsymbol{i} + \sin(3\theta_{2}-2\varphi)\mathbf{j}$(S12)

Addition of the two waves results in a third wave which might be thought to represent the SH field across the interface between the two regions

$\mathbf{P}\sim[sin \left( 3\theta_{1}-2\varphi\right)+\sin\left( 3\theta_{1}-2\varphi\right)]\boldsymbol{i} + [cos \left( 3\theta_{1}-2\varphi\right)+\cos(3\theta_{1}-2\varphi)]\mathbf{j}$(S13)

Making use of the following trigonometric identities

$$\sin\theta+ \sin\varphi=2 sin \left( \frac{\theta+\varphi}{2} \right)\cos\left( \frac{\theta-\varphi}{2} \right)$$

$\cos\theta+ \cos\varphi=2 cos \left( \frac{\theta+\varphi}{2} \right)\cos\left( \frac{\theta-\varphi}{2} \right)$(S14)

we obtain for the total field at the interface

$\mathbf{P}\sim\left[ 2 cos \left( \frac{3}{2}{[\theta}_{1}+\theta_{2}]-2\varphi\right)\cos\left( \frac{3}{2}{[\theta}_{1}-\theta_{2}] \right) \right]\boldsymbol{i} + \left[ 2 sin \left( \frac{3}{2}{[\theta}_{1}+\theta_{2}]-2\varphi\right)\cos\left( \frac{3}{2}{[\theta}_{1}-\theta_{2}] \right) \right] \boldsymbol{j}$ (S15)

For an analyzer perpendicular to the incident beam ($\zeta=\pi/2$ in Eq.1) the total field vanishes due to destructive interference at the interface, when the following conditions for the difference or the sum of the two armchair angles hold

$\theta_{1}-\theta_{2}= \frac{\pi}{3}\left( 4k\pm1 \right)$ (S16)

or

$\theta_{1}+\theta_{2}=\frac{4}{3}\left( \varphi-k\pi-\frac{n \pi}{2} \right)$(S17)

For $k=0,1,2,3\ldots$ and $n=0,1 (n\leq k).$Note that whereas the first condition does not depend on the angle of incident polarization the second condition does and indeed this is what one observes in the experiment. There exist neighboring triangles where the SH signal is zero at their boundary for all polarization angles and others where the signal becomes zero only for specific choices of the polarization angle. In the latter case one can selectively cancel the SH signal by choosing a suitable polarization angle that satisfies the above condition. In addition, the SH field vanishes for any value of difference in the armchair directions which is odd multiple of $\pi/3$.


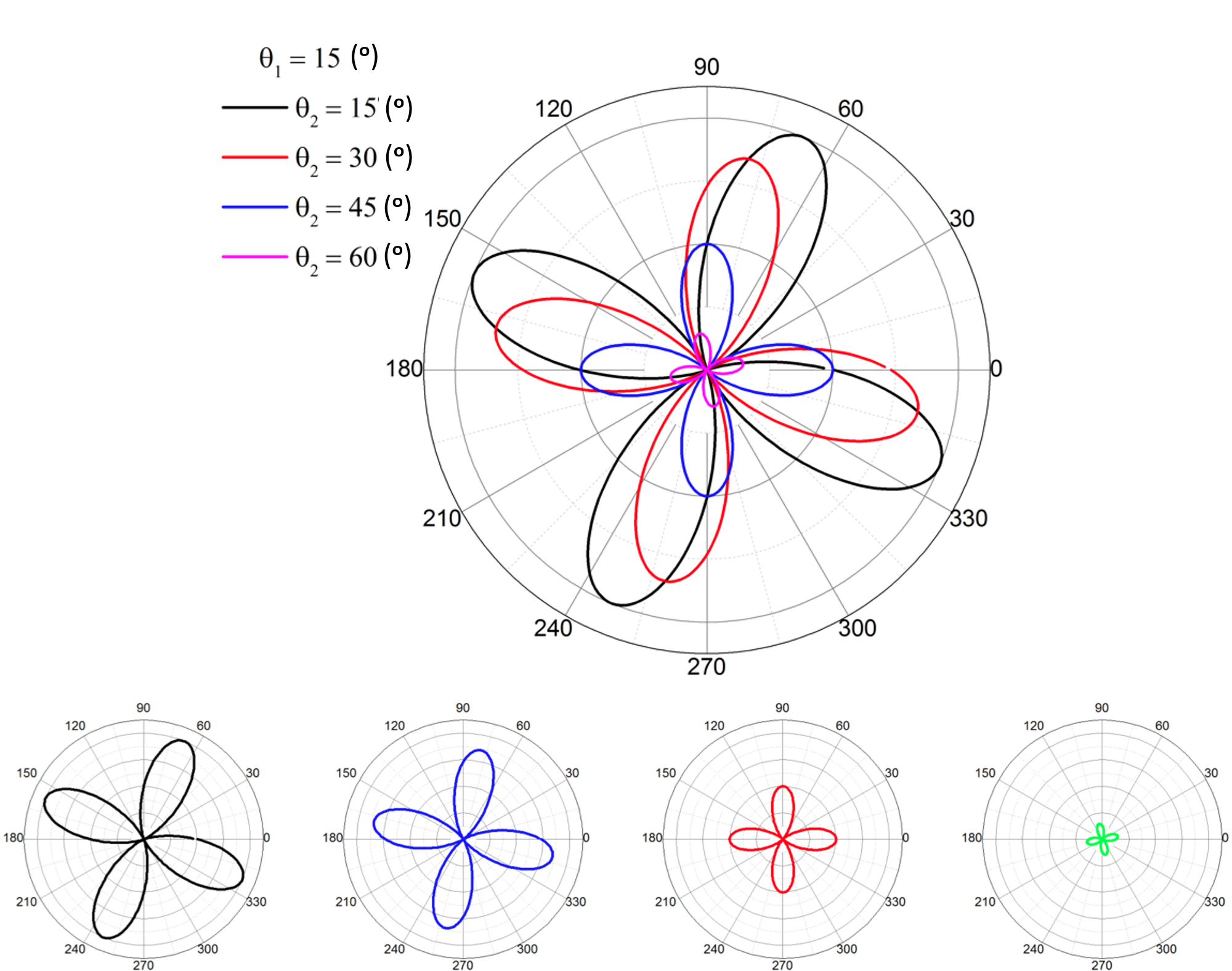


**Figure S3:** Polar diagrams of the SHG intensity at the boundaries between grains with different orientations as a function of the polarization angle. As the difference between the two armchair angles increases, the intensity decreases and the four-leaved rose rotates counter-clockwise.

**3. Raman Analysis and two-photon luminescence (2PL)**

We have used PSHG microscopy for rapidly mapping grain boundaries and orientations, thus determining optically the crystal quality. A direct comparison of the PSHG grain orientation maps from WS_2_ crystals was made against results obtained by Raman spectroscopy and two-photon luminescence (2PL) (Figure S4 and S5).


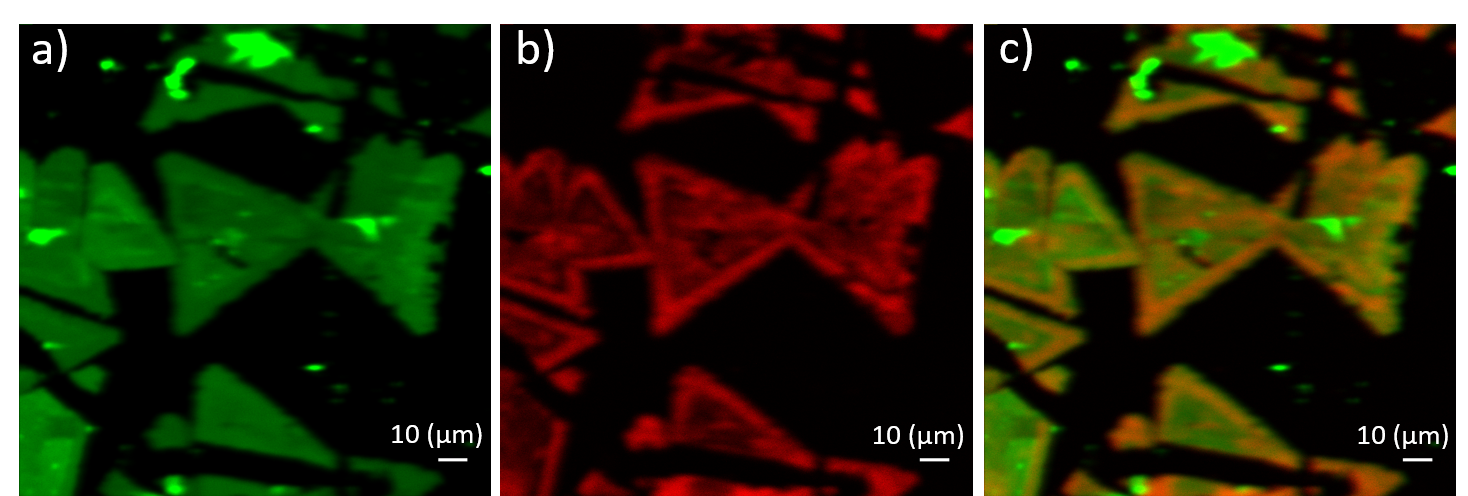


**Figure S4:** (a) Sum of SHG images for excitation polarization orientations *φ* ∈[0°-90°] with step 1°. (b) 2PL imaging of the same region as in (a). (c) Superimposed PSHG and 2PL imaging.

Micro-Raman spectroscopy (Horiba LabRAM HR Evolution), using a 473 nm excitation wavelength with very low intensity in order to avoid structural damage, was employed to identify the number of layers grown by low pressure chemical vapor deposition method (LP-CVD) on a c-cut (0001) sapphire substrate (2D Semiconductors). Regions of randomly distributed WS_2_ flakes were identified with an optical microscope and confirmed with Raman spectroscopy. The laser beam was focused down to 0.6 μm on the sample, placed on an XYZ electronic translation stage at normal incidence. In a typical experiment the Raman system was set in confocal imaging mode, with high precision, allowing nano-step mapping.

Figure S5(b) shows the A_1g_ mode intensity mapping of the excited CVD WS_2_ flake. It is well established that the absolute intensity of the A_1g_ mode increases with increasing the number of layers [3], thus revealing thickness variations across the surface with higher plateaus near their central areas. To further confirm the thickness inhomogeneity across the surface of the WS2 sample in Fig. S5 (c), (d) we have monitored the energy difference between the two most prominent Raman vibrational modes, the fingerprint of the number of layers [3].

**
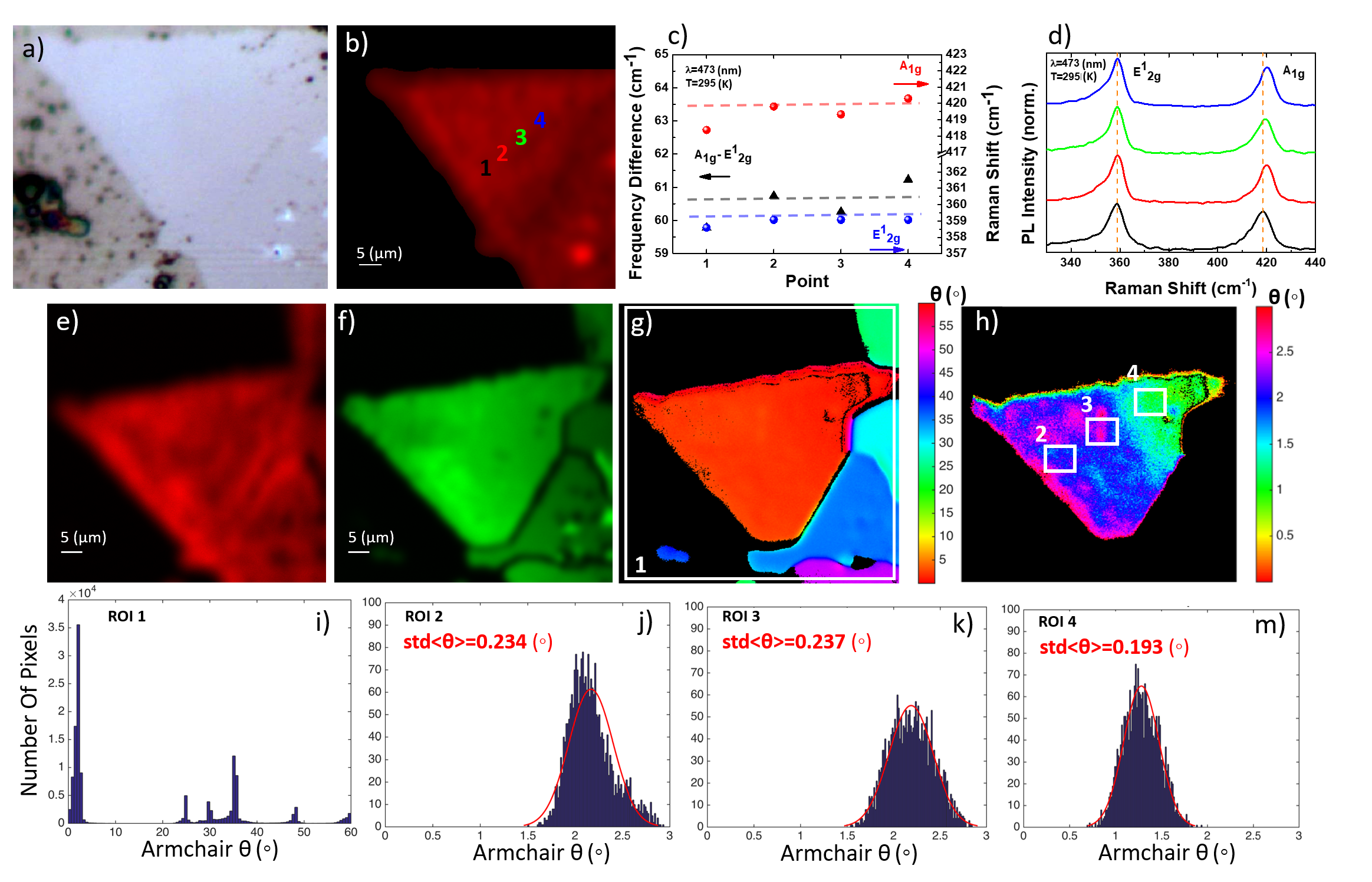
**

**Figure S5**: a) Optical image of WS_2_ on sapphire substrate. b) 473 nm Raman intensity mapping of A_1g_ mode. c) A_1g_ mode’s position for the 4 different points indicated on the Raman image on the left (1 to 4). There is no clear blue shift from point 1 to 4. This indicates a uniform thickness across the WS_2_ surface. d) Raman spectra of the 4 different points indicated on the Raman image on the left (1 to 4); the intensity of the A_1g_ mode does not show significant enhancement, suggesting uniform number of layers. Imaging of the same WS_2_ monolayer using various spectroscopic techniques: e) Two-photon photoluminescence (2PL). f) SHG. g, h) Pixel-by-pixel mapping of WS_2_ crystal orientation (armchair) for 0°≤ *θ* ≤ 60° and 0°≤ *θ* ≤ 3°, respectively. i) Image histogram for the region of interest (ROI) 1. ROI 1 is the rectangle containing the whole image, seen in (g). j-m) Image histograms for the ROIs 2-4, respectively, seen in (h). We note that armchair orientation mapping, using PSHG, offers contrast that is not available with optical, Raman, SHG, 2PL, intensity only based imaging microscopy techniques. The narrow widths for the standard deviations of armchair orientations inside ROIs 2-4 indicate crystal of good quality.

*
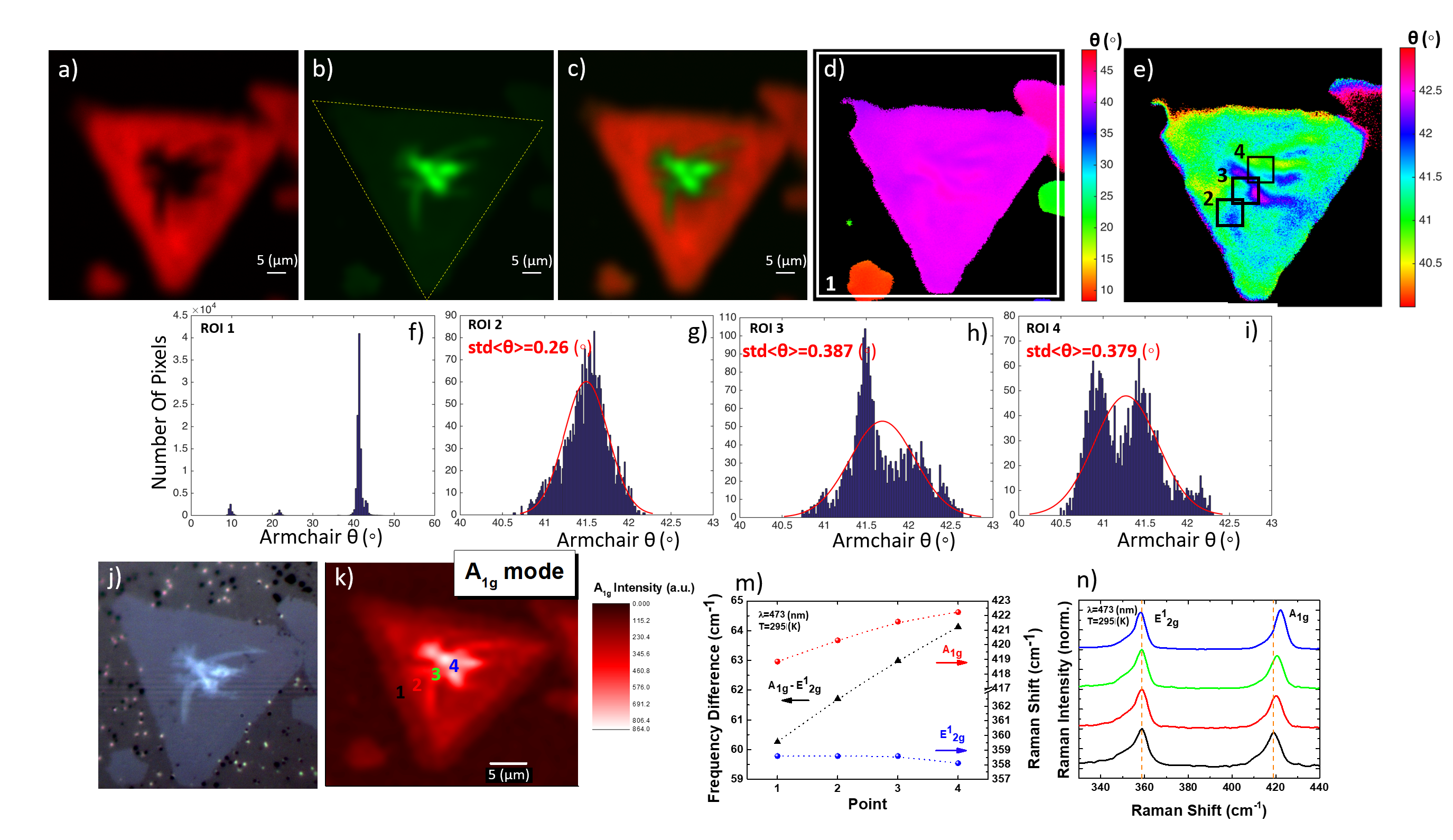
*

**Figure S6:** a) Two-photon photoluminence (2PL). b) SHG imaging of the same WS_2_. c) Combined 2PL and SHG imaging, providing different but complementary information. d,e) Pixel-by-pixel mapping of WS_2_ crystal orientation (armchair). f) Image histogram for the region of interest (ROI) 1, seen in (d). g-i) Image histograms for the ROIs 2-4, respectively, seen in (e). j) Optical image of WS_2_ on sapphire substrate. k) 473 nm Raman intensity mapping of A_1g_ mode. m) A_1g_ mode’s position for the 4 different points indicated on the Raman image on the right (1 to 4). There is clear blueshift from point 1 to 4. This indicates a non uniform thickness across the WS_2_ surface. n) Raman spectra of the 4 different points indicated on the Raman image on the right (1 to 4). We note that the intensity of the A_1g_ mode shows enhancement, which suggests non uniform number of layers. The narrower the width for the standard deviations of armchair orientations, the better the crystals quality is.

Figure S6 shows another comparative imaging of a different triangle while S6 (k)-(n) is the Raman intensity mapping of A_1g_ in the same triangle, where the signal is significantly enhanced. This enhancement is also evident in the SHG image S6(b) and it originates from the increased number of layers in that region. In Fig. S6(k) the peripheral areas display monolayer behavior with energy differences of ~ 60 cm^-1^, the inner regions undoubtedly correspond to multilayered WS_2_ exhibiting energy differences of 64 cm^-1^ (Fig. S6(m)).

**4. Pixel-by-pixel mapping of the armchair direction**

The pixel-by-pixel fitting of the imaging data to the corresponding theoretical model for the crystal structure allows the extraction of detailed information of the boundaries of the defects, due to the change of the armchair crystal direction. The characteristics of the fitting distributions to the theoretical model, such as the mean value or the standard deviation, are exploited as new quantitative optical markers for evaluating the crystal quality of the two-dimensional materials. This concept forms the basis of our novel proposal for ultra-high-resolution mapping of grain orientations. Our results prove the concept of the above methodology in the case of WS_2_ monolayers.

**
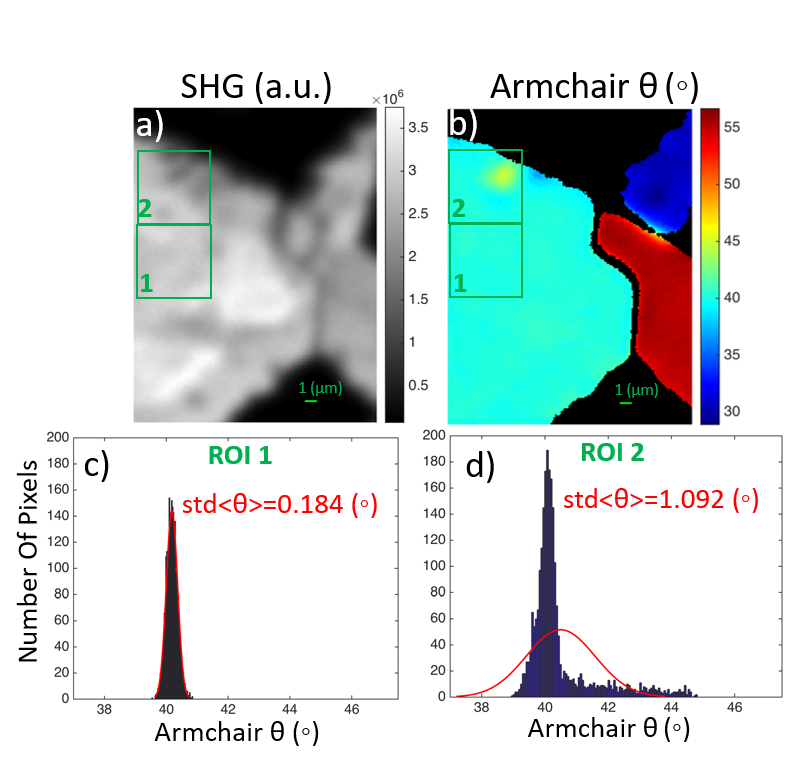
**

**Figure S7:** a) Laser scanning SHG imaging microscopy of CVD grown WS_2_ islands on sapphire substrate. b) Pixel-by-pixel mapping of WS_2_ crystal orientations (armchair *θ*) Two different regions of interest (ROI) are marked. A region of different color, thus different crystal orientations, is easily distinguished in ROI2. c) Image histogram of crystal orientations *θ* for ROI1. Mean Standard deviation of armchair angles *θ*, std<*θ*>=0.184. Narrow crystal orientations distribution indicates a ROI of good quality. d) Image histogram of crystal orientations *θ* for ROI2, std<*θ*>=1.092. In ROI2 the pixels that deviate from the mean value *θ*, result a broader distribution of crystal angles. This is reflected on the bigger value for the std<*θ*>, found for ROI2 in comparison to ROI1. The grain with different crystal orientations, seen in ROI2 compromises the crystal quality, reflected on std<*θ*>.

In Fig. S7 we focus on two regions of interest (ROI), ROI 1 with good crystal quality (narrow standard deviation for the distribution of armchair angles *θ*, (std<*θ*>=0.184) and ROI 2 with moderate crystal quality (std<*θ*>=1.092), due to the presence of pixels with armchair values of multiple orientations.

**5. Determination of the smallest detectable angle change**

In order to determine the smallest angle θ change that is detectable, we performed a simulation using “ideal data” of a WS_2_ PSHG intensity modulation, subject only to the unavoidable shot noise (Poisson distribution is commonly used to describe the effect of photon noise in both non photon counting and photon counting photomultiplier tubes). In particular, using a fixed angle for armchair orientation, we generated N “ideal WS_2_ PSHG” values (number of PSHG measurements) from Eq. 1. In those values, we applied a series of realizations of Poisson noise and performed the same iterative algorithm used to fit the real data. In this way, we extracted the angle θ, for N number of measurements (*φ* ∈[0°, 360°], step 360°/N and *φ* ∈[0°, 90°], step 90°/N). The standard deviation value σ_θ_ that resulted from each set of realizations of Poisson noise, for each number of measurements was utilized as standard error for the experimentally retrieved angles *θ* (σ_θ_ = error[*θ*]). Thus, we deduce that the smallest angle *θ* change that is detectable by the presented PSHG sampling, *φ* ∈[0°, 360°], is estimated numerically to be of the order of, error[*θ_s_*]~ 0.19° (see Fig. S8). This result is very close to the experimental error[*θ_s_*]= 0.17° seen in Figs. 5d)ii,iv obtained for a good crystal quality lattice within a 50x50 pixel ROI.


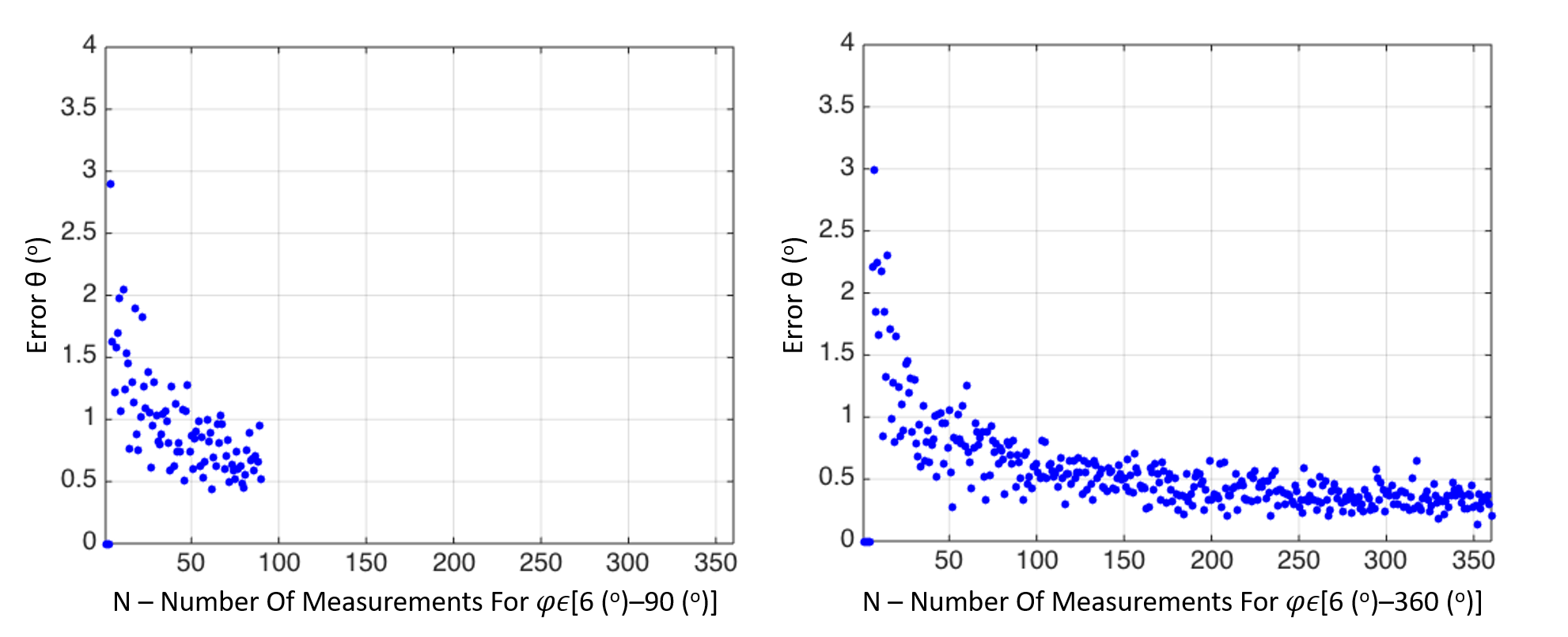


**Figure S8:** Numerical simulation of PSHG minimum achievable angle change. (Left) Min error[*θ_s_*] for *φ* ∈[0°,90°], *θ_s_* = 0.44°, (Right) Min error[θ*_s_*] for *φ* ∈[0°,360°], *θ_s_* = 0.19°.

**6. Precision analysis of PSHG intensity measurements**

We analyzed the estimation precision in the presence of additive Gaussian noise following the procedure described in Ref.[4]. Let $\chi= \left\{ I\left( \varphi_{1} \right), I\left( \varphi_{2} \right),\ldots,I\left( \varphi_{Q} \right) \right\}$ denote the set of SHG intensity measurements $I\left( \varphi_{j} \right)$ performed at $Q$ angles of linear incident polarizations. These SHG intensity measurements are perturbed with an additive Gaussian noise $n\left( \varphi\right)$ with variance $\sigma^{2}$ and zero mean, such that $I\left( \varphi_{i} \right)=I_{0}\left( \varphi_{i} \right)+n(\varphi_{i})$, where $I_{0}\left( \varphi\right)$ the intensity in the absence of noise.

In our case $I_{0}\left( \varphi\right)$ can be written as

$I_{0}\left( \varphi\right)= \alpha_{0}+\alpha_{1}\cos\left[ 2\left( 3\theta-2\varphi\right) \right]$*,*

where the armchair angle $\theta$ defines the orientation of the crystal with respect to the laboratory x-axis and $\alpha_{0}, \alpha_{1}$are related to the second order susceptibility tensor components that characterize the 2D crystal.

It is convenient to introduce the transpose of the vector parameter $\boldsymbol{p}^{T}$= ($p_{1},p_{2},p_{3}$) =($\theta,\alpha_{0},\alpha_{1}$). In PSHG imaging, the parameter $\boldsymbol{p}$ is obtained by fitting $I\left( \varphi\right)$ with iterative algorithms. Let $\boldsymbol{p}$ denote the true parameter’s value and the vector $\hat{\boldsymbol{p}}$(χ) an estimation obtained from the measurement set χ, which can be characterized by its bias $\left\langle\hat{\boldsymbol{p}}(\chi) \right\rangle-\boldsymbol{p}$, and its covariance matrix $\boldsymbol{\Gamma}_{p}=$ $\left\langle\delta\hat{\boldsymbol{p}}(\chi) \delta{\hat{\boldsymbol{p}}(\chi)}^{T} \right\rangle$ with $\delta\hat{\boldsymbol{p}}\left( \chi\right)= \hat{\boldsymbol{p}}$(χ)$- \left\langle\hat{\boldsymbol{p}}(\chi) \right\rangle$. Using statistical estimation theory [5], the covariance matrix $\boldsymbol{\Gamma}$ of any unbiased estimator must satisfy the Cramer-Rao bound (CRB): $\boldsymbol{\nu}^{T} \boldsymbol{\Gamma} \boldsymbol{\nu} \boldsymbol{\geq} \boldsymbol{\nu}^{T} \boldsymbol{I}_{F}^{-1} \boldsymbol{\nu}$for any real vector $\boldsymbol{\nu}$. The Fisher information matrix is $\left[ \boldsymbol{I}_{F} \right]_{nm}=-\left\langle\frac{\partial^{2}l(\chi|\boldsymbol{p})}{\partial p_{n} \partial p_{m}} \right\rangle$, where $l(\chi|\boldsymbol{p})$ is the logarithm of the likelihood $\chi$ given that the parameter is $\boldsymbol{p}$. In the presence of zero mean additive independent Gaussian noise, the logarithm likelihood takes the following form

$l(\chi|\boldsymbol{p})$= $-\sum_{j=1}^{Q} \frac{\left[ I\left( \varphi_{j} \right)-I_{0}\left( \varphi_{j} \right) \right]^{2}}{2 \sigma^{2}} -\frac{1}{2} Q\ln\left[ 2\pi\sigma^{2} \right]$

It is then straightforward to show that $\left[ \boldsymbol{I}_{F} \right]_{nm}= \frac{1}{\sigma^{2}}$ $\sum_{j=1}^{Q} \frac{\partial I_{0}\left( \varphi_{j} \right)}{\partial p_{n}} \frac{\partial I_{0}\left( \varphi_{j} \right)}{\partial p_{m}}$ with $\frac{\partial I_{0}\left( \varphi_{j} \right)}{\partial\theta}=$ 6 $\alpha_{1}$ $\sin\left[ 2\left( 3\theta-2\varphi_{j} \right) \right]$ and $\frac{\partial I_{0}\left( \varphi_{j} \right)}{\partial\alpha_{n}}=\cos\left[ 2n\left( 3\theta-2\varphi_{j} \right) \right]$, for n=0, 1. When the PSHG intensity measurements are performed for $P$ evenly spaced angles $\varphi_{k}$ such that $N$ statistically independent measurements are performed for each angle, we have that $\varphi_{k}=2\pi k/P$ and $Q=PN$. The Fisher information matrix is independent of $\theta$ if $\notin℘=\left\{ 1,2,3,4,6,8 \right\}$ [5] and it therefore has nonzero elements only in the diagonal. In particular, for $P=360$ and $N=1$ the diagonal elements of the Fisher information matrix are respectively equal to $6480 a_{1}^{2}/\sigma^{2}$, $360/\sigma^{2}$, $180/\sigma^{2}$. Consequently, the variances of the parameters satisfy

$$\left\langle\left[ \hat{\theta}\left( \chi\right)-\theta\right]^{2} \right\rangle\geq\frac{\sigma^{2}}{6480a_{1}^{2}}$$

$$\left\langle\left[ \hat{\alpha_{n}}\left( \chi\right)-\alpha_{n} \right]^{2} \right\rangle\geq A_{n}\frac{\sigma^{2}}{360}$$

with $A_{0}=1$and $A_{1}=2.$It is evident that the precision depends only on the product $\mathrm{PN}$ as long as $P\notin℘.$ Thus, using this noise model, the same precision can be achieved by performing 10 measurements for each 36 evenly spaced angles or if one measurement is performed each 360 evenly spaced angles. Because the off-diagonal terms of the Fisher information matrix are equal to zero, there is no correlation between the measured parameters if $P\notin℘.$ Thus, knowledge of the $\alpha_{n}$ parameters would not improve the precision of estimation of $\theta$.

In our experiment, we have chosen to use a step of 1o in $\varphi$ and perform one measurement for each angle instead of performing many measurements at each angle with a larger step. This choice allows a faster convergence of the iterative fitting algorithm. Note however that, according to the above analysis, the step in the polarization angle of the incident beam does not influence the accuracy of the armchair angle estimation as long as $P\notin℘.$

Nevertheless, in order to be able to obtain information from individual pixels of interest, a large number of measurements is necessary and although this increases the duration of the experiment, it reduces dramatically the time needed for the fitting algorithm to converge. Using the above inequalities we estimate a minimum variance of 0.05 degrees in $\theta$.

**7. Pixels in regions of destructive interference**

In Fig. S9b, the SHG signal in the cancelation region is very weak in intensity and below the threshold for good fitting. The threshold value can be used to filter erroneous pixels and to create additional contrast not present in the SHG intensity image. The filtering effect is demonstrated in Fig. S9c where only the pixels exhibiting quality of fitting, as low as R^2^> 0.1, survive during the fitting procedure. As a result, a black line, corresponding to absence of armchair angle information, is generated in the armchair image.

***
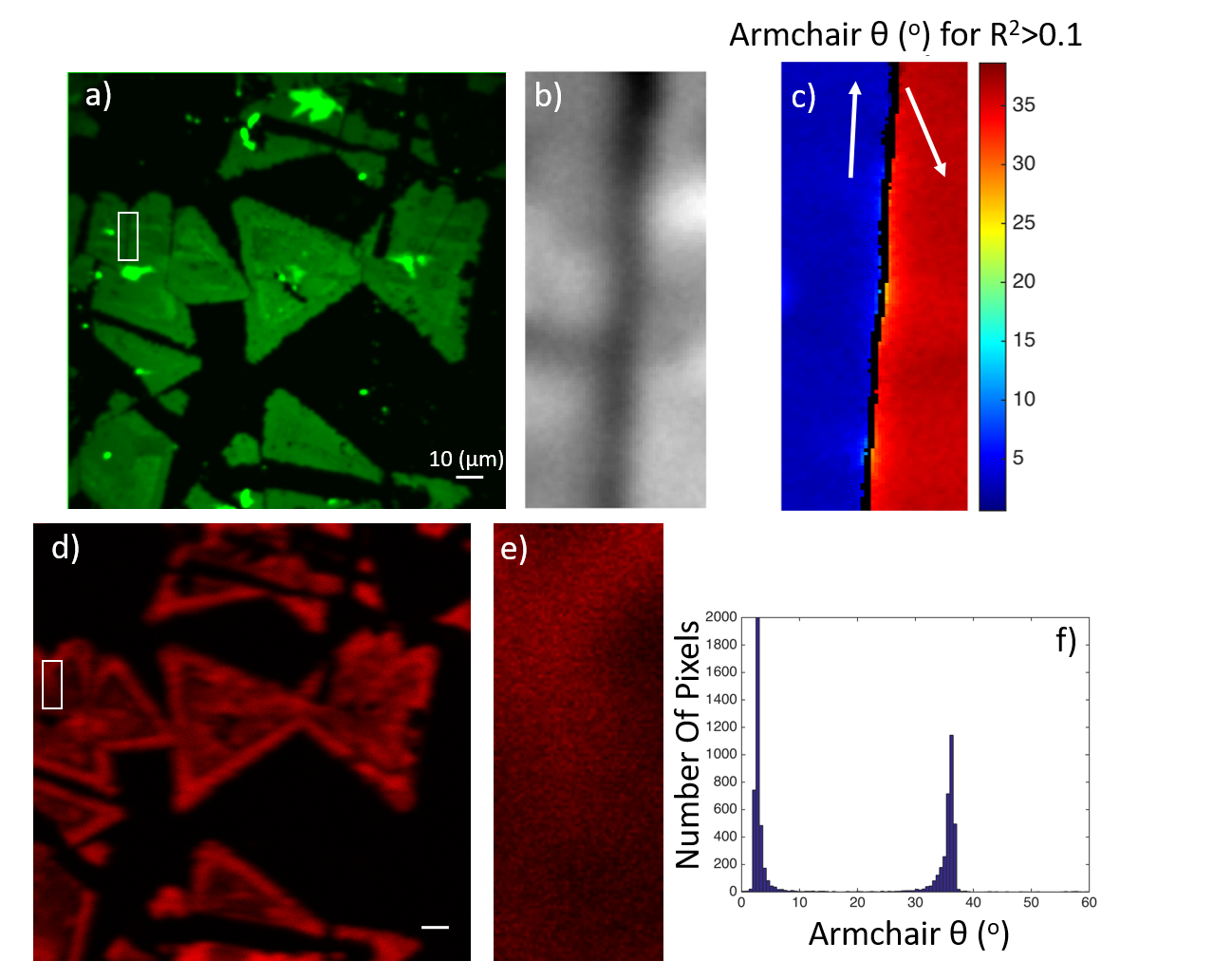
***

**Figure S9: a**) Integrated SHG intensity for *φ* ∈[0^o^- 90^o^], b) Integrated SHG intensity for ROI seen in (a) for *φ* ∈[0^o^-360^o^], c) Armchair mapping of region seen in (b) for R^2^>0.1. The armchair contrast expands inside the region of lower SHG signal without providing observable additional intermediate *θ* values inside the cancelation region. d) 2PL image of same region seen in (a), e) 2PL of the ROI seen in (a, d) and (b). It is obvious that lack of SHG seen in (b) is not due to lack in material, since 2PL image seen in (e) is homogenous. f) Image histogram of the armchair values *θ* of the ROI seen in (a-e).

Note that in Fig. S9b, the small SHG signal in the edges of the cancelation line did not affect the retrieval of the armchair value (see Fig. S9c and video 3), providing sub-diffraction discrimination between regions of different crystal orientations. Any additional intermediate *θ* values are not observed inside the cancelation region.

**Video Captions**

**Video 1:** Forward detected polarization resolved SHG intensity imaging of WS_2_ islands on sapphire substrate (Brighter color indicates higher PSHG intensity). Double arrows show the orientation *ζ* of the analyzer (arrows, upper right) and the orientation *φ* (*φ* ∈ [1°-360°], step 1°) of the linear excitation polarization (arrows, lower right).

**Video 2:** Pixel-wise fitting of the experimental data for a raster-scanned (from left to right) region between the POI 1 and POI 3 seen in Fig. 6a and Fig. 6b. In the video we note that the four-leaved rose rotates, depending on the armchair direction contained in every pixel. Although the change in the armchair direction is small (*θ* is approximately between 30° and 40°), in Fig. 6b we note 2 distinct regions of different contrast (light blue to dark blue), in the continuous crystal lattice (compare Fig. 6a with Fig. 6b, concentrate on the interface between light blue and dark blue in Fig. 6b). This sensitivity in the identification of small changes in the armchair orientation *θ*, allows to create contrasted regions in a continuous 2D crystal layer, which are not seen in the intensity only SHG image (compare Fig. 6a with Fig. 6b in the interface between light blue and dark blue in Fig. 6b).

**Video 3**: Real time raw data and the result of the fitting in the ROI seen in Fig. S9.

**References**

[1] Psilodimitrakopoulos S, Santos SICO, Amat-Roldán I, Nair ATK, Artigas-García D, Loza-Alvarez P, editors. In vivo, pixel-resolution mapping of thick filaments' orientation in nonfibrilar muscle using polarization-sensitive second harmonic generation microscopy. J. Biomed. Opt.;2009: **14**(1), 014001.

[2] Hristu R, Stanciu SG, Tranca DE, Polychroniadis EK, Stanciu GA. Identification of stacking faults in silicon carbide by polarization-resolved second harmonic generation microscopy. Scientific Reports. 2017;**7**(1):4870.

[3] Berkdemir A, Gutiérrez HR, Botello-Méndez AR, Perea-López N, Elías AL, Chia C-I, et al. Identification of individual and few layers of WS2 using Raman Spectroscopy. Scientific Reports. 2013;**3**:1755.

[4] Réfrégier P, Roche M, Brasselet S. Precision analysis in polarization-resolved second harmonic generation microscopy. Opt Lett. 2011;**36**(11):2149-51.

[5] Poor HV, An introduction to signal detection and estimation. Verlag: Springer, 1998.
